# Sebetralstat for breakthrough attacks in patients with hereditary angioedema receiving long-term prophylaxis in KONFIDENT-S

**DOI:** 10.1016/j.jacig.2026.100750

**Published:** 2026-06-12

**Authors:** Tamar Kinaciyan, Emel Aygören-Pürsün, Inmaculada Martinez-Saguer, Daniel F. Soteres, Daisuke Honda, Michael E. Manning, H. James Wedner, Atsushi Fukunaga, Henry J. Kanarek, Kazumasa Ohmura, Isaac Melamed, Tariq El-Shanawany, Laurence Bouillet, William R. Lumry, Marc A. Riedl, Majed Koleilat, Bob Geng, Isao Ohsawa, Noemi Bara, Syed Rehman, Jonathan A. Bernstein, Paula J. Busse, Mauro Cancian, Danny M. Cohn, Timothy J. Craig, Henriette Farkas, Sorena Kiani-Alikhan, H. Henry Li, Jason P. Raasch, Raffi Tachdjian, Paul K. Audhya, Paolo Bajcic, Ya-Hsiu Chuang, Matthew Iverson, Michael D. Smith, Christopher M. Yea, Andrea Zanichelli

**Affiliations:** a Department of Dermatology, ACARE and Hereditary Angioedema Center Vienna and Burgenland, Medical University of Vienna, Vienna, Austria; b University Hospital Frankfurt, Goethe University, Frankfurt, Germany; c HZRM Haemophilia Center Rhein Main, Frankfurt, Germany; d Asthma & Allergy Associates PC, Colorado Springs, Colo; e Department of Nephrology, Graduate School of Medicine, Chiba University, Chiba, Japan; f Department of Internal Medicine, UA College of Medicine, Phoenix, Ariz; g Washington University School of Medicine, St Louis, Mo; h Department of Dermatology/OMPU Allergy Center, Division of Medicine for Function and Morphology of Sensory Organs, Faculty of Medicine, Osaka Medical and Pharmaceutical University, Osaka, Japan; i Kanarek Adult & Pediatric Allergy, Asthma & Immunology, Overland Park, Kan; j Institute of Preventive Medical Sciences, Health Sciences University of Hokkaido, Sapporo, Japan; k IMMUNOe Research Center, Centennial, Colo; l Department of Immunology, University Hospital of Wales, Cardiff, United Kingdom; m University of Grenoble Alpes, T-RAIG Unit, CNRS, UMR 5525, CHU Grenoble Alpes, TIMC, Grenoble, France; n French National Reference Center for Angioedema (CREAK), Internal Medicine Department, Grenoble University Hospital, Grenoble, France; o AARA Research Center, Dallas, Tex; p University of California–San Diego, La Jolla, Calif; q Deaconess Clinic, Evansville, Ind; r Allergy Asthma Medical Group & Research Center (AAMGRC), San Diego, Calif; s Department of Nephrology, Saiyu Soka Hospital, Saitama, Japan; t Hereditary Angioedema Expertise Center, Sangeorgiu de Mures, Romania; u Mediquest Clinical Research Center, Sangeorgiu de Mures, Romania; v Toledo Institute of Clinical Research, Toledo, Ohio; w University of Cincinnati College of Medicine and Bernstein Clinical Research Center, Cincinnati, Ohio; x Division of Allergy and Clinical Immunology, Icahn School of Medicine at Mount Sinai, New York, NY; y Departmental Allergy Division, Department of Systems Medicine, University of Padua, Padua, Italy; z Department of Vascular Medicine, Amsterdam Cardiovascular Sciences, Amsterdam University Medical Center, University of Amsterdam, Amsterdam, The Netherlands; aa Department of Medicine, Pediatrics, MFM and Biomedical Sciences, Penn State University, Hershey, Pa; bb Vinmec-VinUni Institute of Immunology, Vin University, Hanoi, Vietnam; cc Center of Allergy and Clinical Immunology, Vinmec Times City Hospital, Hanoi, Vietnam; dd Hungarian Angioedema Center of Reference and Excellence, Department of Internal Medicine and Haematology, Semmelweis University, Budapest, Hungary; ee Division of Infection and Immunity, University College London, Royal Free London NHS Foundation Trust, London, United Kingdom; ff Institute for Asthma and Allergy, Wheaton, Md; gg Midwest Immunology Clinic, Plymouth, Minn; hh Division of Allergy and Clinical Immunology, David Geffen School of Medicine, University of California, Los Angeles, Los Angeles, Calif; ii Allergy and Immunology, Providence Saint John’s Health Center, Santa Monica, Calif; jj KalVista Pharmaceuticals, Framingham, Mass; kk Operative Unit of Medicine, Angioedema Center, IRCCS Policlinico San Donato, San Donato Milanese, Milan, Italy; ll Dipartimento di Scienze Biomediche per la Salute, University of Milan, Milan, Italy

**Keywords:** Berotralstat, C1 inhibitor, hereditary angioedema, lanadelumab, long-term prophylaxis, on-demand treatment, sebetralstat

## Abstract

**Background:**

Although long-term prophylaxis (LTP) reduces attack frequency in hereditary angioedema, patients may experience breakthrough attacks. Oral sebetralstat demonstrated favorable safety and efficacy compared with placebo in the randomized phase 3 KONFIDENT trial (NCT05259917), including in patients receiving LTP. The safety and effectiveness of sebetralstat in participants receiving LTP are being assessed in the KONFIDENT-S open-label extension study (NCT05505916).

**Objective:**

This interim analysis of the KONFIDENT-S study evaluated long-term safety and effectiveness of oral sebetralstat 600 mg for attacks of hereditary angioedema with C1-inhibitor deficiency in participants receiving LTP with lanadelumab, berotralstat, or C1 inhibitor.

**Methods:**

Efficacy end points included times to beginning of symptom relief, reduction in severity, and complete attack resolution.

**Results:**

As of September 14, 2024, 35 participants receiving LTP experienced a mean ± standard deviation of 1.7 ± 1.5 attacks per month and treated 382 attacks with sebetralstat (1.3 ± 1.1 sebetralstat-treated attacks per month). Median (interquartile range) time from attack recognition to sebetralstat administration was 6 (1-40) minutes; time to beginning of symptom relief was 1.3 (0.8 to 3.8) hours, time to reduction in severity was 4.2 (1.3 to >12) hours, and time to complete attack resolution was 14.8 (4.6 to >24) hours. Effectiveness was similar for participants receiving berotralstat, lanadelumab, or C1 inhibitor. No serious or severe treatment-related treatment-emergent adverse events were reported.

**Conclusion:**

Sebetralstat was well tolerated and enabled early on-demand treatment of attacks in patients with hereditary angioedema with C1-inhibitor deficiency receiving LTP. Treatment of breakthrough attacks with sebetralstat resulted in rapid symptom relief, reduction in attack severity, and complete attack resolution, regardless of LTP mechanism of action.

Hereditary angioedema with C1-inhibitor deficiency (HAE-C1INH) is a rare genetic disease that results in uncontrolled activation of the kallikrein–kinin system (KKS), which manifests clinically as unpredictable and potentially painful and debilitating attacks of tissue swelling.^1^^,^^2^ Rarely, attacks can become life-threatening if they lead to airway compromise^3^ or circulatory collapse.^4^ On-demand treatment is aimed to reduce morbidity and prevent the mortality associated with hereditary angioedema (HAE) attacks.^1^ In patients with HAE-C1INH, early administration of on-demand treatment has been shown to shorten attack duration and reduce the time to attack resolution.^5^, ^6^, ^7^, ^8^ On-demand treatments approved by the US Food and Drug Administration and/or the European Medicines Agency are sebetralstat (an oral plasma kallikrein inhibitor),^9^^,^^10^ icatibant (a subcutaneous bradykinin B2 receptor antagonist),^11^^,^^12^ ecallantide (a subcutaneous plasma kallikrein inhibitor that must be administered by a health care professional),^13^ and plasma-derived or recombinant human C1INH (intravenous C1INH replacement).^14^, ^15^, ^16^, ^17^, ^18^

For patients with HAE-C1INH who are already receiving on-demand treatment, guidelines recommend that long-term prophylaxis (LTP) be considered on the basis of the disease activity, patient quality of life, availability of health care resources, and failure to obtain adequate control by on-demand treatment alone.^2^ Targeted LTP agents act to inhibit the KKS by several mechanisms, including direct inhibition of plasma kallikrein activation (ie, lanadelumab^19^^,^^20^ and berotralstat^21^^,^^22^), direct inhibition of factor XIIa and transformation of prekallikrein into plasma kallikrein (ie, garadacimab^23^^,^^24^), inhibition of prekallikrein production (ie, donidalorsen^25^), or C1INH protein replacement^16^^,^^26^, ^27^, ^28^ to increase the concentration of functional C1INH (which in turn inhibits both the conversion of factor XII to factor XIIa and the activation of plasma kallikrein).^29^

A 2024 systematic review of clinical studies evaluating LTP agents showed that many patients continued to experience breakthrough attacks of all severities and in all anatomic locations, including the larynx.^30^ The efficacy of LTP agents in reducing attack frequency appears to be dependent on consistent maintenance of a plasma concentration that results in a level of inhibition of the KKS that mimics healthy physiology.^31^^,^^32^ Therefore, rather than being related to the specific mechanism of individual LTP agents, breakthrough attacks likely occur when the plasma concentration of the LTP agent decreases below a minimum concentration threshold necessary to maintain adequate inhibition of plasma kallikrein.^32^, ^33^, ^34^, ^35^, ^36^, ^37^, ^38^ Most breakthrough attacks in clinical studies were treated with at least one dose of on-demand medication; however, little has been reported regarding the effectiveness of on-demand treatment in patients receiving LTP.^30^

The clinical development program for sebetralstat, the most recently approved on-demand treatment, allowed the concomitant use of approved targeted LTP therapies.^39^^,^^40^ The randomized, double-blind, phase 3 KONFIDENT trial (NCT05259917) included 24 participants receiving targeted LTP (stable dose and regimen for at least 3 months before random assignment) who treated 58 attacks with study drug.^39^ There were no notable differences in safety and effectiveness of sebetralstat in participants receiving LTP and those receiving only on-demand treatment to manage HAE-C1INH.^39^

The objective of this prespecified interim analysis of the 2-year multicenter KONFIDENT-S open-label extension study (NCT05505916)^40^ is to report the safety and effectiveness of oral sebetralstat in treating a much larger number of breakthrough attacks than in the KONFIDENT trial in patients receiving concurrent LTP with berotralstat, lanadelumab, or C1INH.

## Methods

### Study design and participants

The KONFIDENT-S study design and eligibility criteria have been previously described.^40^ The study protocol was approved by institutional review board/ethics committees, and the study is being conducted in accordance with the principles of Good Clinical Practice as described by the International Conference on Harmonization, applicable local regulatory and legal requirements, and the principles of the Declaration of Helsinki. All participants provided written informed consent or assent.

Eligible participants were adults and adolescents ≥12 years of age with HAE-C1INH who experienced at least 2 documented attacks within 3 months before enrollment, or those who had completed the phase 3 KONFIDENT randomized controlled trial. Participants receiving LTP with lanadelumab, berotralstat, or plasma-derived C1INH were required to maintain a stable dose and regimen for ≥3 months before enrollment. Following a protocol amendment after study initiation, participants were permitted to start, stop, or change LTP agents during the trial at the discretion of the principal investigator.

Consistent with global treatment guidelines,^1^^,^^2^ participants were instructed to self-administer treatment (ie, sebetralstat 600 mg) as early as possible after attack onset, regardless of the severity or anatomic location of the attack at the time of treatment. An optional second administration of sebetralstat was allowed ≥3 hours after the first administration, if determined to be necessary by the participant. Conventional treatment with an approved on-demand medication (ie, icatibant, recombinant human or plasma-derived C1INH, or ecallantide [United States only]) was allowed at any time if warranted, as determined by the participant, including as initial treatment for an attack.

### Primary and secondary objectives

The primary objective in the KONFIDENT-S study was safety, assessed by adverse event reporting and supplemented by laboratory test results, vital signs, and physical examination. The secondary objective, effectiveness, was assessed by the time to beginning of symptom relief within 12 hours (defined as a Patient Global Impression of Change response of at least “a little better” for ≥2 consecutive time points), time to reduction in attack severity within 12 hours (defined as ≥1 level decrease on the Patient Global Impression of Severity [PGI-S] for ≥2 consecutive time points), and time to complete attack resolution within 24 hours (defined as a PGI-S response of “none”). The time to end of attack progression was analyzed *post hoc* and was defined as the time at which the worst attack severity was recorded on the PGI-S scale within 12 hours after administration of the first dose of sebetralstat. Effectiveness data were only collected for attacks treated with on-demand sebetralstat first and were not collected for attacks treated with a conventional on-demand medication first.

Quality of life was assessed through a symptom-specific health-related instrument for patients with recurrent angioedema (AE-QoL), consisting of 17 questions across the 4 domains of functioning, fatigue/mood, fears/shame, and food, to evaluate the extent of angioedema-dependent quality-of-life impairment during the previous 28 days. Each AE-QoL question had 5 answer options (scored 1 to 5), with higher scores indicating a greater adverse impact on quality of life. The total score from the AE-QoL was then transformed into a linear scale ranging from 0 to 100, with a higher score indicating greater impairment in quality of life. Participants completed the AE-QoL at baseline and every 28 ± 7 days thereafter through the end of the trial.

Treatment satisfaction for sebetralstat-treated attacks was assessed at 24 hours after administration for each attack using a 7-point scale ranging from extremely dissatisfied (−3) to extremely satisfied (+3).

### Statistical analysis

Data were summarized descriptively; categorical data were reported by participant counts and percentages. Continuous data were reported using descriptive statistics (eg, mean, standard deviation [SD], median, interquartile range [IQR], range, hazard ratio, least squares [LS], mean, and confidence interval [CI]). Attacks were right-censored at 0 hours if a time to event result could not be derived. Analysis of time to beginning of symptom relief allowed missing data entries between consecutive time points. Administration of conventional on-demand treatment was censored to the end of the analysis window. Change from baseline in the LS mean (95% CI) for overall AE-QoL and individual AE-QoL domain scores was calculated from a mixed-effects model with repeated measures, using fixed effects of baseline and month and taking into consideration correlation of scores from the same participant. All analyses were performed by SAS v9.4 or later (SAS Institute, Cary, NC).

## Results

### Participants

**Fig 1:**
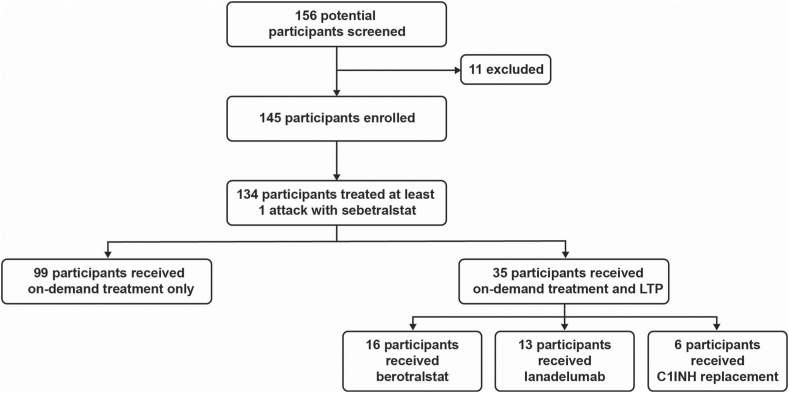
Study CONSORT (Consolidated Standards of Reporting Trials) diagram.

From the beginning of enrollment on October 21, 2022, to the data cutoff date of September 14, 2024, 134 participants administered sebetralstat for at least one attack (Fig 1), of which 35 participants (26.1%) had been receiving LTP with berotralstat (n = 16), lanadelumab (n = 13), or C1INH (n = 6) (Table I). Four participants who received LTP at baseline switched to a different LTP agent during the study: 1 participant switched from C1INH LTP to lanadelumab and was included in the lanadelumab group, 1 participant switched from C1INH to berotralstat and was included in the berotralstat group, 1 participant switched from lanadelumab to C1INH and was included in the lanadelumab group, and 1 participant switched from berotralstat to C1INH and was included in the berotralstat group. No participants who were receiving LTP at baseline discontinued LTP during the study; 1 of 99 participants who received on-demand treatment only at baseline initiated LTP during the study and was not included in the current analysis.

**Table I:** Participant demographics and disease-specific history

Characteristic	Participants receiving any LTP agent	Participants receiving berotralstat	Participants receiving lanadelumab	Participants receiving C1INH LTP
No. of participants	35	16	13	6
Age (years), median (IQR)	44.0 (28.0-56.0)	38.5 (21.0-48.0)	44.0 (31.0-60.0)	48.5 (28.0-54.0)
Age group				
≥12 to <18 years	5 (14.3)	4 (25.0)	1 (7.7)	0
≥18 to <65 years	29 (82.9)	12 (75.0)	12 (92.3)	5 (83.3)
≥65 years	1 (2.9)	0	0	1 (16.7)
Sex				
Female	27 (77.1)	13 (81.3)	11 (84.6)	3 (50.0)
Male	8 (22.9)	3 (18.8)	2 (15.4)	3 (50.0)
Race				
Asian	8 (22.9)	4 (25.0)	3 (23.1)	1 (16.7)
White	25 (71.4)	10 (62.5)	10 (76.9)	5 (83.3)
Other	1 (2.9)	1 (6.3)	0	0
Not reported	1 (2.9)	1 (6.3)	0	0
Region				
North America	19 (54.3)	7 (43.8)	7 (53.8)	5 (83.3)
Europe	9 (25.7)	5 (31.3)	3 (23.1)	1 (16.7)
Asia-Pacific	7 (20.0)	4 (25.0)	3 (23.1)	0
Body mass index (kg/m^2^), median (IQR)	26.6 (22.1-33.1)	27.1 (21.6-33.8)	25.3 (24.2-27.3)	32.1 (30.6-37.7)
HAE-C1INH type				
Type 1	31 (88.6)	15 (93.8)	13 (100)	3 (50.0)
Type 2	4 (11.4)	1 (6.3)	0	3 (50.0)

Data are presented as nos. (%) unless otherwise indicated. LTP agents included berotralstat, lanadelumab, and C1INH replacement. Four participants who had been receiving LTP at baseline switched to different LTP agent during study: 1 participant switched from C1INH replacement to lanadelumab and was included in lanadelumab group, 1 participant switched from C1INH replacement to berotralstat and was included in berotralstat group, 1 participant switched from lanadelumab to C1INH replacement and was included in lanadelumab group, and 1 participant switched from berotralstat to C1INH replacement and was included in berotralstat group.

Most participants receiving LTP were female (77.1%), were White (71.4%), and had a diagnosis of type 1 HAE-C1INH (88.6%) (Table I). The median (IQR) age was 44.0 (28.0-56.0) years, and 5 participants (14.3%) were adolescents (aged 12 to <18 years at enrollment). Most participants were from North America (19, 54.3%), followed by Europe (9, 25.7%) and Asia-Pacific (7, 20.0%). Participant enrollment per country is reported in Table E1 in the Online Repository available at www.jaci-global.org.

### Attack frequency and treatment patterns

Participants receiving LTP experienced a total of 504 breakthrough attacks, and the mean ± SD overall frequency was 1.7 ± 1.5 attacks per month: 1.8 ± 1.4 for participants receiving berotralstat, 1.2 ± 1.1 for participants receiving lanadelumab, and 2.5 ± 2.2 for participants receiving C1INH LTP. The median (IQR) overall frequency was 1.0 (0.6-2.7) attacks per month: 1.2 (0.6-3.4), 0.8 (0.7-1.0), and 2.1 (1.2-2.7) for berotralstat, lanadelumab, and C1INH LTP, respectively. Of the 504 breakthrough attacks, 496 (98.4%) were treated with on-demand treatment: 382 (75.8%) were treated initially with sebetralstat, and 114 (22.6%) were treated initially with a parenterally administered on-demand medication. Eight attacks (1.6%) remained untreated.

### Treated attack characteristics

Participants receiving berotralstat, lanadelumab, and C1INH LTP treated 178, 80, and 124 attacks with sebetralstat, respectively. The mean ± SD frequency of sebetralstat-treated attacks per month was 1.3 ± 1.1 for all participants receiving LTP (median [IQR], 0.8 [0.5-1.8]), 1.4 ± 1.2 for participants receiving berotralstat (0.9 [0.4-2.0]), 0.8 ± 0.5 for participants receiving lanadelumab (0.7 [0.5-0.8]), and 2.0 ± 1.4 for participants receiving C1INH LTP (1.9 [1.2-2.3]).

Baseline attack severity, location, and time to treatment of sebetralstat-treated attacks are reported in Table II. Overall, 29.6% of breakthrough attacks were mild at the time of treatment. Participants receiving lanadelumab treated a numerically lower proportion of severe or very severe attacks with sebetralstat (7.5%) compared with participants receiving berotralstat (29.8%) or C1INH LTP (28.2%). Approximately 50% of sebetralstat-treated breakthrough attacks were mucosal (ie, attacks with primary location of abdomen and/or larynx/throat), including 17 attacks that involved the larynx comprising 8 in participants receiving berotralstat, 7 receiving lanadelumab, and 2 receiving C1INH LTP.

**Table II:** Baseline attack characteristics of sebetralstat-treated HAE-C1INH attacks in participants receiving LTP

Characteristic	Attacks in participants receiving:			
	Any LTP agent	Berotralstat	Lanadelumab	C1INH LTP
No. of attacks	382	178	80	124
Baseline PGI-S category∗				
Mild	112 (29.3)	58 (32.6)	24 (30.0)	30 (24.2)
Moderate	141 (36.9)	64 (36.0)	48 (60.0)	29 (23.4)
Severe/very severe	94 (24.6)	53 (29.8)	6 (7.5)	35 (28.2)
Missing	34 (8.9)	2 (1.1)	2 (2.5)	30 (24.2)
Primary pooled attack locations†				
Mucosal	189 (49.5)	107 (60.1)	55 (68.8)	27 (21.8)
Abdominal only	128 (33.5)	79 (44.4)	39 (48.8)	10 (8.1)
Abdominal and subcutaneous	44 (11.5)	20 (11.2)	9 (11.3)	15 (12.1)
Laryngeal	17 (4.5)	8 (4.5)	7 (8.8)	2 (1.6)
Subcutaneous only	159 (41.6)	69 (38.8)	23 (28.8)	67 (54.0)
Missing	34 (8.9)	2 (1.1)	2 (2.5)	30 (24.2)
Time (minutes) from attack onset to treatment, median (IQR)	6 (1-40)	20 (1-67)	11 (1-50)	1 (0-7)

Data are presented as nos. (%) unless otherwise indicated. LTP agents included berotralstat, lanadelumab, and C1INH replacement.

∗ One attack in participant who received LTP with berotralstat (0.6%) reported an attack severity of none.

† *Mucosal* refers to attacks with primary location of abdomen and/or larynx/throat (subcutaneous involvement possible); *laryngeal,* attacks involving the larynx/throat (abdominal and subcutaneous involvement possible); and *subcutaneous only,* other attacks not involving mucosal locations.

For the 114 attacks (22.6% of total attacks) initially treated with conventional injectable therapy, the proportion that were reported as mild or moderate at the time of treatment was 62.3% of attacks, which was comparable to 66.2% of attacks initially treated with sebetralstat. The proportion of attacks involving a mucosal site (ie, abdominal or laryngeal) was also similar for attacks treated with conventional on-demand therapy first (43.9%) and sebetralstat first (49.5%). However, the proportion of laryngeal attacks was higher, at 19.3% (22 attacks) among attacks treated with conventional on-demand first versus 4.5% (17 attacks) among those treated with sebetralstat first. There were no demographic differences between the 21 participants who administered conventional on-demand first at least once (median age, 45 years; 76% female) and the full cohort of 35 participants (median age, 44 years; 77% female).

Median (IQR) time from attack recognition to sebetralstat administration was 6 (1-40) minutes. Time from attack onset to administration of sebetralstat varied somewhat by LTP agent, with a median (IQR) of 1 (0-7) minute reported for participants receiving C1INH LTP, 11 (1-50) minutes for those receiving lanadelumab, and 20 (1-67) minutes for those receiving berotralstat.

### Safety

Of the 35 participants receiving LTP, 23 (65.7%) experienced treatment-emergent adverse events (TEAEs) (Table III). TEAEs in 5 participants (14.3%) were considered by investigators to be related to treatment with sebetralstat: headache (berotralstat, 1; C1INH LTP, 2), myalgia (berotralstat, 1), arthralgia (berotralstat, 1), nausea (berotralstat, 1), and vomiting (berotralstat, 1).

**Table III:** Safety in participants receiving LTP who treated ≥1 attack with sebetralstat

Participants	Participants receiving any LTP agent	Participants receiving berotralstat	Participants receiving lanadelumab	Participants receiving C1INH LTP
No. of participants	35	16	13	6
Any TEAE	23 (65.7)	12 (75.0)	6 (46.2)	5 (83.3)
**Treatment related**	**5 (14.3)**	**3 (18.8)**	**0**	**2 (33.3)**
Serious TEAE∗	5 (14.3)	3 (18.8)	1 (7.7)	1 (16.7)
**Treatment related**	**0**	**0**	**0**	**0**
Severe TEAE†	7 (20.0)	3 (18.8)	2 (15.4)	2 (33.3)
**Treatment related**	**0**	**0**	**0**	**0**
Any TEAE leading to sebetralstat discontinuation	2 (5.7)	1 (6.3)	1 (7.7)	0
**Treatment related**‡	**1 (2.9)**	**1 (6.3)**	**0**	**0**
Any TEAE leading to death	0	0	0	0

LTP agents included berotralstat, lanadelumab, and C1INH replacement.

∗ Serious TEAE was defined as any untoward medical occurrence that at any dose resulted in death, was life-threatening, required inpatient hospitalization or prolongation of existing hospitalization, resulted in persistent or significant disability/incapacity, was congenital anomaly/birth defect, or was important medical event by medical and scientific judgment.

† Baseline severe (grade 3 or 4) TEAEs were evaluated by investigators according to Toxicity Grading Scale for Healthy Adult and Adolescent Volunteers Enrolled in Preventive Vaccine Clinical Trials.

‡ One participant who received berotralstat discontinued sebetralstat because of treatment-related TEAEs of grade 2 nausea and grade 2 vomiting, which occurred during attack involving abdomen and larynx/throat.

One participant receiving lanadelumab discontinued sebetralstat because of a TEAE of increased alanine aminotransferase level; this TEAE was not considered related to sebetralstat but was attributed to a serious adverse event of viral meningitis (which was secondary to herpes simplex 2) in combination with a comorbid autoimmune condition with concomitant receipt of several potentially hepatoxic medications. One participant receiving berotralstat discontinued sebetralstat because of TEAEs of grade 2 nausea and grade 2 vomiting, which occurred during an attack that involved the abdomen and larynx/throat. No serious or severe sebetralstat-related TEAEs were reported in any participants. No clinically significant trends after sebetralstat treatment were seen in laboratory test results, vital sign assessments, electrocardiograms, or physical examination.

### Effectiveness

Median (IQR) time to beginning of symptom relief was 1.3 (0.8-3.8) hours for participants receiving any LTP and also 1.3 (0.8-2.5) hours for participants receiving kallikrein-inhibiting LTP (berotralstat or lanadelumab). Median (IQR) time to reduction in attack severity and time to complete attack resolution, respectively, was 4.2 (1.3 to >12) hours and 14.8 (4.6 to >24) hours for participants receiving any LTP; and 3.3 (1.1 to >12) hours and 12.1 (3.4 to >24) hours for participants receiving kallikrein-inhibiting LTP. Data by individual LTP agent are presented in Table IV. Median (IQR) time to end of progression within 12 hours for participants receiving any LTP was 20.4 (16.8-40.8) minutes, 18.0 (16.2-27.6) minutes for those receiving berotralstat, 24.0 (16.8-58.8) minutes for those receiving lanadelumab, and 26.4 (16.8-61.2) minutes for those receiving C1INH LTP. Of the 346 attacks for which data were available, 315 (91.0%) reached end of progression within 4 hours (96.5% for berotralstat, 90.8% for lanadelumab, and 81.6% for C1INH LTP). Among attacks treated with sebetralstat, effectiveness was generally consistent between participants receiving LTP and participants receiving on-demand treatment only: The hazard ratios (95% CI) for time to beginning of symptom relief, time to reduction in severity, and time to complete resolution were 1.38 (0.93-2.04), 1.53 (0.98-2.40), and 1.75 (1.03-2.99), respectively (see Fig E1 in the Online Repository available at www.jaci-global.org).

**Table IV:** Time to symptom relief within 12 hours, time to reduction in attack severity within 12 hours, and time to complete attack resolution within 24 hours in sebetralstat-treated breakthrough attacks

Attacks in participants receiving:	No. of participants, n	Sebetralstat-treated attacks, n	Median (IQR) time (hours) to:		
			Beginning of symptom relief∗	Reduction in attack severity†	Complete resolution‡
Any LTP agent	35	382	1.3 (0.8 to 3.8)	4.2 (1.3 to >12)	14.8 (4.6 to >24)
Kallikrein-inhibiting LTP (berotralstat or lanadelumab)	29	258	1.3 (0.8 to 2.5)	3.3 (1.1 to >12)	12.1 (3.4 to >24)
Berotralstat	16	178	1.3 (0.4 to 2.5)	2.7 (0.9 to >12)	10.9 (3.0 to >24)
Lanadelumab	13	80	1.3 (0.8 to 2.7)	4.4 (1.4 to >12)	15.1 (3.7 to >24)
C1INH LTP	6	124	1.8 (1.3 to >12)	>12 (1.8 to >12)	16.6 (9.0 to 23.5)

∗ Defined as a Patient Global Impression of Change rating of at least “a little better” for 2 consecutive time points, with missing data entries between consecutive time points included.

† Defined as the time to first incidence of decrease from baseline in PGI-S score for at least 2 consecutive time points within 12 hours of first dose of sebetralstat.

‡ Defined as a PGI-S rating of “none” (ie, no symptoms) within 24 hours of first dose of sebetralstat.

**Fig 2:**
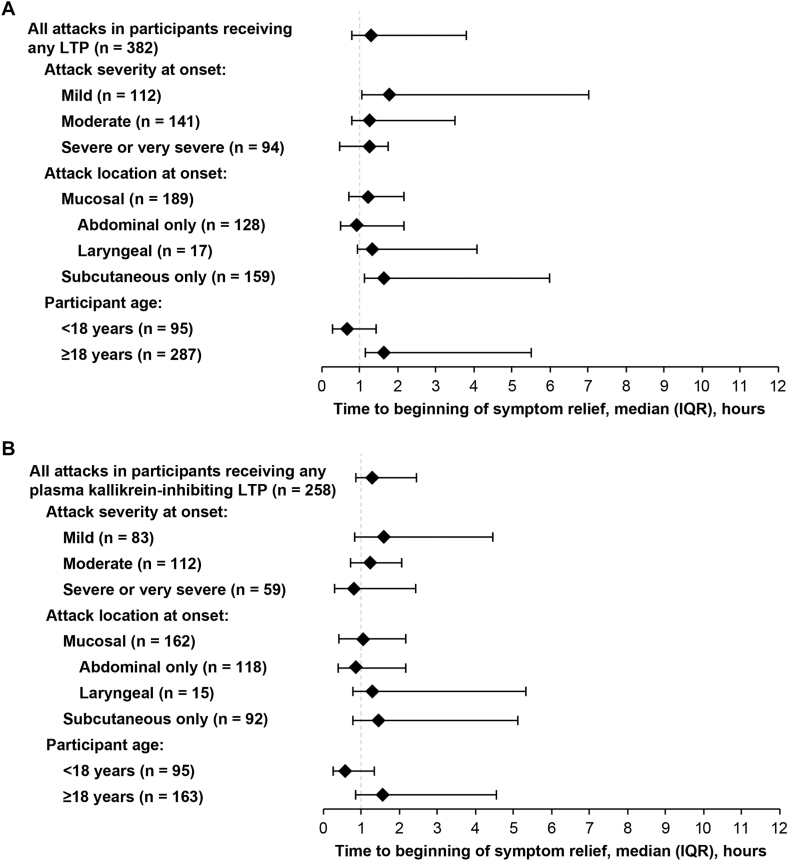
Subgroup analysis of time to beginning of symptom relief by participant age group, attack severity at time of sebetralstat administration, and attack location in participants receiving **(A)** any LTP agent and **(B)** LTP with plasma kallikrein–inhibiting agent (berotralstat or lanadelumab). *Diamonds* are medians; *error bars,* Q1 and Q3.

In subgroup analyses by attack severity at the time of sebetralstat administration, median (IQR) times to beginning of symptom relief in participants receiving any LTP agent were 1.8 (1.0-7.0) hours, 1.3 (0.8-3.5) hours, and 1.3 (0.5-1.8) hours for mild, moderate, and severe/very severe attacks, respectively; results were comparable for participants receiving a plasma kallikrein–inhibiting LTP agent (berotralstat or lanadelumab) (Fig 2, *A* and *B*).

Subgroup analyses by attack location showed median (IQR) times to beginning of symptom relief in participants receiving any LTP agent of 1.3 (0.7-2.2) hours, 1.4 (0.9-4.1) hours, 1.0 (0.5-2.2), and 1.7 (1.2-6.0) hours for attacks with mucosal involvement, attacks with laryngeal involvement, attacks limited to the abdomen, and attacks limited to subcutaneous tissues, respectively; results were comparable for participants receiving a plasma kallikrein–inhibiting LTP agent (Fig 2, *A* and *B*).

In subgroup analyses by age, median (IQR) times to beginning of symptom relief in participants receiving any LTP agent were 0.7 (0.3-1.4) hours and 1.7 (1.1-5.5) hours for participants aged <18 years (n = 5) and participants aged ≥18 years (n = 30), respectively; results were comparable for participants receiving a plasma kallikrein–inhibiting LTP agent (Fig 2, *A* and *B*).

A second dose of sebetralstat was administered within 12 hours in 22.3% of attacks (21.3%, 11.3%, and 30.6% of attacks in participants receiving berotralstat, lanadelumab, and C1INH LTP, respectively). Of the 382 attacks, 264 (69.1%) obtained beginning of symptom relief within 12 hours. Of these, 90.5% obtained the end point without or before a second dose of sebetralstat. Among participants receiving a plasma kallikrein–inhibiting LTP agent, 77.5% of attacks obtained beginning of symptom relief within 12 hours, and 92.5% of these did so without or before a second dose of sebetralstat. Conventional on-demand treatment within 12 hours was administered in 20 sebetralstat-treated attacks (5.2%): 16 administrations (80%) occurred after the first dose of sebetralstat and 4 administrations (20%) occurred after the second dose of sebetralstat. Participants receiving berotralstat, lanadelumab, and C1INH LTP administered conventional on-demand treatment within 12 hours in 8 (4.5%), 5 (6.3%), and 7 (5.6%) breakthrough attacks, respectively. Among participants receiving on-demand treatment only, conventional on-demand treatment within 12 hours was administered in 76 (5.7%) of 1324 sebetralstat-treated attacks.

### Other patient-reported outcomes

**Fig 3:**
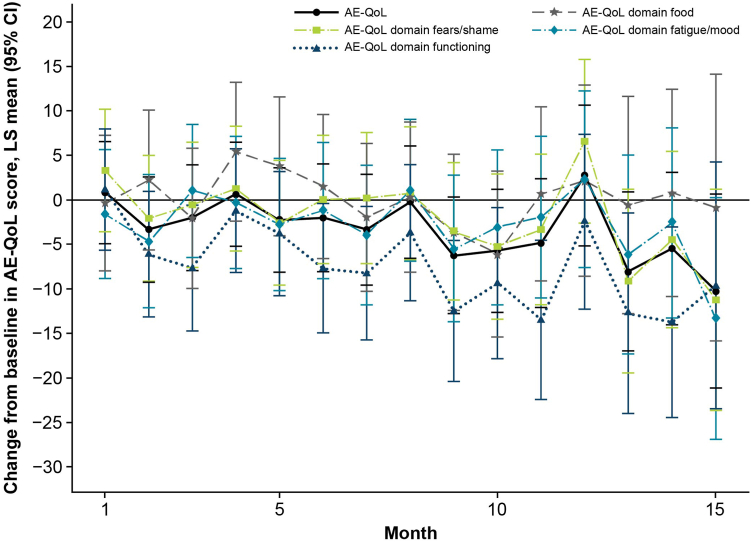
LS mean (95% CI) change in overall AE-QoL scores and in individual domains of functioning, fatigue/mood, fears/shame, and food, from baseline through month 15 in participants receiving LTP.

AE-QoL scores were available for 34 participants receiving LTP. AE-QoL scores from baseline to month 15 tended to improve over time, with a LS mean (95% CI) change from baseline of −10.3 (−21.1 to 0.6) at month 15 (Fig 3). Mean scores for each of the AE-QoL domains, except food, showed a decrease at month 15: Changes in LS mean (95% CI) from baseline were −9.6 (−23.5 to 4.2), −13.3 (−26.9 to 0.2), and −11.2 (−23.6 to 1.3) for functioning, fatigue/mood, and fears/shame, respectively.

Treatment satisfaction scores were available for 28 participants receiving LTP and for a total of 276 sebetralstat-treated attacks. Of these, participants reported being satisfied with sebetralstat treatment (rating of at least +1) for 245 attacks (88.8%), and very satisfied (+2) or extremely satisfied (+3) for 207 attacks (75.0%).

## Discussion

The unpredictable nature of breakthrough attacks in patients receiving LTP underscores the ongoing need for effective on-demand therapies.^30^ Although several injectable on-demand treatments have been approved by regulators globally,^29^^,^^41^^,^^42^ parenteral administration is associated with a substantial treatment burden that makes it hard for patients to adhere to treatment guidelines, and it often leads to suboptimal clinical outcomes due to delayed or withheld treatment.^43^^,^^44^ Sebetralstat is the first oral on-demand treatment for HAE attacks in adult and pediatric patients 12 years of age and older^9^^,^^10^ and has the potential to eliminate many of the barriers that prevent timely administration of on-demand treatment.^29^ This prespecified analysis of the KONFIDENT-S open-label extension study provides compelling evidence that sebetralstat is generally well tolerated and effective across various LTP regimens, attack severities, and anatomic locations. Given the larger cohort of patients receiving LTP and a substantially larger number of breakthrough attacks than were treated in the KONFIDENT trial,^39^ these results inform a more comprehensive understanding of the potential role of sebetralstat in this common clinical scenario.

In this analysis, sebetralstat was used to treat an average of 1.3 breakthrough attacks per month, with the highest frequency reported in participants receiving C1INH LTP and the lowest in those receiving lanadelumab. Attacks of all severities and in all anatomic locations, including the larynx, occurred in participants receiving LTP. The median time from attack onset to sebetralstat administration was 6 minutes, which may reflect the protocol-based instruction for participants to treat attacks as early as possible. Notably, 29.6% of attacks were treated while still mild, before progression. While these observations do not predict real-world treatment behaviors with certainty, they suggest that oral on-demand treatment enabled patients to adhere to treatment guidelines to treat early after attack recognition.^1^^,^^2^ Indeed, in a recently published Delphi study on the barriers to on-demand treatment of HAE attacks, there was 100% consensus on the statement: “On-demand HAE therapies that have fewer side effects and are easier to store, carry, and administer may increase adherence to guidance on treating HAE attacks early.”^45^

Of the 504 breakthrough attacks that occurred in participants receiving LTP, only 1.6% were left untreated. This contrasts with only 50% of attacks treated with an approved injectable on-demand medication in a large prospective observational study of 227 patients (4270 attacks) conducted before the availability of sebetralstat.^6^ It was notable in that study that the proportion of attacks treated with an approved parenteral on-demand medication was inversely proportional to attack severity, highlighting that patients were more likely to treat attacks that had progressed to severe (>60% treated) and less likely to treat attacks that were still mild (<40% treated).^6^

In line with the LTP subgroup in the phase 3 KONFIDENT trial,^39^ sebetralstat provided early symptom relief, followed quickly by reduction in severity and symptomatic resolution. Additional subgroup analyses affirmed the broad effectiveness of sebetralstat: No substantial numerical differences were observed according to attack severity or anatomic location. Crucially, among participants receiving plasma kallikrein–inhibiting LTP (ie, lanadelumab or berotralstat), time to beginning of symptom relief was similarly rapid, which did not substantiate concerns that sebetralstat might not be as effective for attacks in patients receiving on-demand treatment and LTP with the same mechanisms of action.

Of interest, the time to end of progression within 12 hours was reached in a median of 20.4 minutes for participants receiving LTP. This finding further suggests that sebetralstat halts the worsening of attack symptoms shortly after dosing and drug absorption. Further analysis showed that 91% of sebetralstat-treated attacks reached end of progression within 4 hours.

The rate of additional on-demand treatment was low; 22.3% of attacks were treated with a second dose of sebetralstat within 12 hours, and 5.2% of attacks were treated with conventional on-demand medication within 12 hours. Among the 20 attacks that received conventional on-demand treatment within 12 hours, 16 were administered after the first dose of sebetralstat, while only 4 were administered after a second dose of sebetralstat. Overall, these rates indicate that a single dose of sebetralstat was sufficient to obtain therapeutic goals for most breakthrough attacks. Indeed, 90.5% of sebetralstat-treated attacks that obtained beginning of symptom relief within 12 hours did so without or before a second dose of sebetralstat.

A unique feature of KONFIDENT-S was that participants were given the option of treating each attack with sebetralstat or their conventional injectable on-demand treatment. Under these conditions, participants opted to treat 77% of attacks with sebetralstat. Consistent with an earlier analysis of KONFIDENT-S data,^40^ the 23% of attacks in which conventional therapy was chosen for treatment generally occurred early in the study, suggesting initial caution followed by progressive adoption of sebetralstat after gaining experience. Interestingly, attacks treated with conventional on-demand medication were not on average more severe; however, they were somewhat more likely to involve the larynx, which may also reflect a circumspect approach.

In a first for an on-demand treatment, AE-QoL scores (evaluated every 28 days) for participants receiving LTP who treated their breakthrough attacks with sebetralstat showed a trend for improvement over time. This trend appeared to be driven primarily by improvements in the functioning domain (ie, work, physical activities, leisure activities, and social relations). Through month 15, the mean overall AE-QoL score and mean scores for all domains except for food (ie, restrictions on eating and drinking) showed a >6 point change, exceeding the minimal clinically important difference for the AE-QoL^46^ and suggesting a meaningful improvement in quality of life. Consistent with these findings, satisfaction for breakthrough attacks treated with sebetralstat was high in participants receiving LTP, with ratings of satisfied, very satisfied, or extremely satisfied for approximately 90% of attacks.

Sebetralstat was well tolerated in KONFIDENT-S, and no new safety signals were observed in participants receiving LTP with berotralstat, lanadelumab, or C1INH. Most TEAEs were rated as mild or moderate, and few TEAEs were considered treatment related by investigators. Only one participant receiving berotralstat discontinued sebetralstat because of adverse events of nausea and vomiting, which occurred during an attack involving the abdomen and larynx. Differentiating gastrointestinal TEAEs from abdominal attack symptoms may be complicated by their symptom overlap.

Limitations of this study include its open-label design, which, although reflecting a real-world treatment environment and allowing for a larger number of breakthrough attacks, introduces the potential for reporting bias. The descriptive nature of the analysis also limits causal inferences. Furthermore, the study was not designed or powered to evaluate differences in the effectiveness of sebetralstat by the specific LTP agent received or differences between subgroups; these data are reported to offer a preliminary clinical overview. The number of participants receiving C1INH LTP in the study was low, and the findings therefore might not be generalizable or representative of this patient subgroup.

In conclusion, results of this interim analysis of the KONFIDENT-S study demonstrate that sebetralstat is a safe, well-tolerated, and effective on-demand treatment for breakthrough attacks in patients receiving LTP, regardless of the LTP mechanism of action. Sebetralstat enabled early treatment, halting attack progression and leading to rapid symptom relief and resolution of attack symptoms, with participants experiencing high levels of treatment satisfaction and potential improvements in quality of life. These data support the role of sebetralstat as an important component of HAE management.

## Disclosure statement

Supported by KalVista Pharmaceuticals, Framingham, Mass.

Disclosure of potential conflict of interest: T. Kinaciyan has received support for the present report, consulting fees, support with article processing fees, honoraria, and/or meeting/travel support from and/or has served on advisory boards and data safety monitoring boards for KalVista Pharmaceuticals, Pharvaris, Takeda, BioCryst, CSL Behring, Otsuka, and Astria. E. Aygören-Pürsün has received grants, consulting fees, and/or honoraria from and has served on advisory boards for KalVista Pharmaceuticals, Astria, BioCryst, BioMarin Europe, CSL Behring, Intellia Therapeutics, Pharming Technologies, Pharvaris, and Takeda/Shire. I. Martinez-Saguer has received grants, royalties or licenses, consulting fees, honoraria, clinical trial support, medical writing support, article processing charges, meeting/travel support, and course sponsorship from and has served on advisory boards and data safety monitoring for KalVista Pharmaceuticals, Takeda, CSL Behring, Pharming, BioCryst, Octapharma, and Pharvaris. D. F. Soteres has received grants, consulting fees, and/or honoraria from KalVista Pharmaceuticals, BioCryst, CSL Behring, Intellia Therapeutics, Ionis Pharmaceuticals, Pharming, Pharvaris, and Takeda. D. Honda has received honoraria from and has served on advisory boards for KalVista Pharmaceuticals, BioCryst, Takeda, Torii Pharmaceutical Co, and CSL Behring; and has a leadership position with HAE Japan (HAEJ) and Diagnostic Consortium to Advance the Ecosystem for Hereditary Angioedema (DISCOVERY). M. E. Manning has received grants, consulting fees, and honoraria and has served on advisory and data safety monitoring boards for KalVista Pharmaceuticals, BioCryst, CSL Behring, Ionis Pharmaceuticals, Pharvaris, Pharming, Takeda, Astria, BioMarin, Cogent, Teva, GSK, Novartis, Allakos, Celldex Therapeutics, Aimmune Therapeutics, Blueprint Medicines, Amgen, AstraZeneca, Sanofi, Genentech, Regeneron, and Intellia Therapeutics. H. J. Wedner has received consulting fees, speaker fees, and/or research funding from KalVista Pharmaceuticals, Takeda, Pharvaris, BioMarin, Astria, Ionis Pharmaceuticals, CSL Behring, Intellia Therapeutics, and Allergy Therapeutics. A. Fukunaga has received consulting fees and honoraria from KalVista Pharmaceuticals, CSL Behring, Takeda, and Torii Pharmaceutical Co; and serves as chair of the Creation Committee for the Japanese Dermatological Association Urticaria Treatment Guidelines, 4th edition. H. J. Kanarek has received honoraria paid to the institution from Pharming and BioCryst. K. Ohmura has received consulting fees and honoraria from Takeda, CSL Behring, and BioCryst. T. El-Shanawany has received educational support, research support, speaker fees, and consultant fees from AliveDX, ALK-Abello, Allergy Therapeutics, BioCryst, CSL Behring, Grifols, KalVista Pharmaceuticals, Octapharma, Novartis, Takeda, and Viatris. L. Bouillet has received consulting fees, honoraria, medical writing support, payment for expert testimony, and meeting/travel support from and has served on advisory and data safety monitoring boards for KalVista Pharmaceuticals, Takeda, CSL Behring, BioCryst, and Pharvaris; and has been vice president of the Société Nationale Française de Médecine Interne (SNFMI). W. R. Lumry has received consulting fees, grants, and research support from KalVista Pharmaceuticals, Astria, BioCryst, BioMarin, CSL Behring, Express Scripts/CVS, Fresenius Kabi, Intellia Therapeutics, Magellan, Optum, Pharming, Pharvaris, Shire/Takeda, Optinose, Grifols, AstraZeneca, Sanofi/Regeneron, GSK, Ionis Pharmaceuticals, and Teva; and has board membership with the US Hereditary Angioedema Association medical advisory board (HAEA) and the DFW Metroplex Allergy Society. M. A. Riedl has received grants, consulting fees, and funding for a clinical trial from KalVista Pharmaceuticals, BioCryst, BioMarin, CSL Behring, Ionis Pharmaceuticals, Pharvaris, Takeda, Astria, Celldex, Cycle Pharma, Grifols, Intellia Therapeutics, Pfizer, Pharming, and Sanofi/Regeneron. M. Koleilat has received honoraria from AstraZeneca. B. Geng has been a speaker for, received consulting fees, and/or received research support from KalVista Pharmaceuticals, Pharming, Takeda, BioCryst, Ionis Pharmaceuticals, and Astra. I. Ohsawa has received honoraria and/or has been a speaker for CSL Behring, Takeda, BioCryst, and Torii Pharmaceutical Co. N. Bara has received honoraria, financial support for conducting research, meeting/travel support, medical writing assistance, and/or served on advisory boards for BioCryst, CSL Behring, Takeda, Pharming, Swixx Biopharma, and Zentiva; is vice president of the Romanian Registry for HAE foundation; is vice president of the Romanian Society of Allergy and Clinical Immunology; and is head of the Romanian Committee for Hereditary Angioedema. S. Rehman has received honoraria and been a speaker for and/or has received funding and clinical trial support from KalVista Pharmaceuticals, AstraZeneca, Takeda, Sanofi, Novartis, Genentech, Teva, Evomune, Celldex Therapeutics, GSK, Octapharma, Kedrion, and Windward Bio. J. A. Bernstein has received grants and honoraria from KalVista Pharmaceuticals, ADARx Pharmaceuticals, Ajou University, Allergy Therapeutics, Amgen, Apogee Therapeutics, Areteia, ARS Pharmaceuticals, AstraZeneca, Astria, BioCryst, Blueprint Medicine, Celldex, Cogent, CSL Behring, Eli Lilly, Escient Pharmaceuticals, Evommune, Fresenius Kabi, Genentech, GSK, Incyte, Intellia Therapeutics, Ionis Pharmaceuticals, Japan Tobacco Company, Jasper Therapeutics, Kenvue, Kymera Therapeutics, Kyowa Kirin, Medscape, Merck, Nasus Pharma, Nektar Therapeutics, Neopharma, Novartis, Opella, Pharming, Pharvaris, Proctor & Gamble, Regeneron, Sanofi, Takeda/Shire, Telios Pharma, Teladoc Health, Teva, Yuhan, and WebMD news; is consultant for Enanta Pharmaceuticals, Pfizer, Proctor & Gamble, and RAPT Therapeutics; is speaker for KalVista Pharmaceuticals, Pharming, CSL Behring, Pharming, and Novartis; is past president of the American Academy of Allergy, Asthma & Immunology (AAAAI); chairs the AAAAI Foundation; and is a member of the HAEA medical advisory board. P. J. Busse has received consulting fees from KalVista Pharmaceuticals, Adarx, Astria, BioCryst, BioMarin, CSL Behring, CVS Specialty, Novartis, Pharvaris, Regeneron, and Takeda; has received payment for expert testimony from Hinkley Allen; and has a leadership role in the HAEA. M. Cancian has received honoraria and meeting/travel support paid to the institution from KalVista Pharmaceuticals, BioCryst, CSL Behring, Pharvaris, and Takeda. D. M. Cohn has received consulting fees paid to the institution, honoraria paid to the institution, medical writing support, meeting/travel support, and research support from and has served on advisory boards for KalVista Pharmaceuticals, Astria, BioCryst, CSL Behring, Intellia Therapeutics, Ionis Pharmaceuticals, Otsuka, Pharvaris, and Takeda; and has had a leadership role in the HAE-international (HAEi) medical advisory panel for Central Eastern Europe and Benelux. T. J. Craig has received grants, consulting fees, and honoraria from and has served on advisory boards and data safety monitoring boards for KalVista Pharmaceuticals, CSL Behring, GSK, Astria, ADARx, Argo, Takeda, BioMarin, BioCryst, Pharming, Ionis Pharmaceuticals, Grifols, Pharvaris, and Intellia Therapeutics; serves as director for Angioedema Centers of Reference and Excellence (ACARE) International Hereditary Angioedema Center and Alpha-1 Resource Center; and is a member of the HAEA medical advisory board. H. Farkas has received grants paid to the institution, honoraria, medical writing support, and meeting/travel support from and has served on advisory boards for KalVista Pharmaceuticals, Astria, BioCryst, CSL Behring, Intellia Therapeutics, Ionis Pharmaceuticals, Ono Pharmaceutical, Pharming, Pharvaris, and Takeda; and has had a leadership role on the ACARE Steering Committee. S. Kiani-Alikhan has received consulting fees, honoraria, medical writing support, and meeting/travel support from and has served on advisory boards and/or data safety monitoring for KalVista Pharmaceuticals, BioCryst, Takeda, CSL Behring, Pharvaris, Astria, Ionis Pharmaceuticals, and Otsuka Pharmaceuticals (UK). H. H. Li has received consulting fees, honoraria, research support, medical writing support, grants or contracts for clinical trials paid to the institution, and/or has served on advisory boards and data safety monitoring boards for KalVista Pharmaceuticals, Argo Biopharma, CSL Behring, BioCryst, Pharming, Takeda, Intellia Therapeutics, Pharvaris, Ionis Pharmaceuticals; and has held a leadership or fiduciary role in the HAEA medical advisory board. J. P. Raasch has received consulting fees, honoraria, clinical trial support from and has served on advisory boards and data safety monitoring boards for KalVista Pharmaceuticals, CSL Behring, Ionis Pharmaceuticals, Pharvaris, Pharming, and Takeda. R. Tachdjian has received grants, advisory and consulting fees, and speaking fees from KalVista Pharmaceuticals, Astria, Ionis Pharmaceuticals, CSL Behring, Takeda, BioCryst, Pharvaris, Grifols, Lilly, Novartis, Pharming, Sanofi, Regeneron, AstraZeneca, Intellia Therapeutics, and GSK. P. K. Audhya, P. Bajcic, and C. M. Yea are former employees and shareholders of KalVista Pharmaceuticals. Y. H. Chuang, M. Iverson, and M. D. Smith are current employees and shareholders of KalVista Pharmaceuticals. A. Zanichelli has received consulting fees, honoraria, meeting/travel support from, and has served on advisory boards for Astria, KalVista Pharmaceuticals, BioCryst, Intellia Therapeutics, CSL Behring, Otsuka, Pharvaris, and Takeda; and has held a leadership or fiduciary role in the Italian Network for Hereditary and Acquired Angioedema (ITACA), Associazione volontaria per l’angioedema ereditario ed altre forme rare di angioedema (AAEE), and Società Italiana di Medicina Interna (SIMI). I. Melamed declares no relevant conflicts of interest.

## Supplementary data

Supplementary Table SI

Supplementary Figure E1
