# Supplementary material for: Sebetralstat for breakthrough attacks in patients with hereditary angioedema receiving long-term prophylaxis in KONFIDENT-S
Source: J Allergy Clin Immunol Glob. 2026 Jun 12;5(5):100750. doi: 10.1016/j.jacig.2026.100750 (PMC13355760; doi:10.1016/j.jacig.2026.100750)
Supplement: Supplementary Table SI [file mmc1.docx]

**TABLE SI.** Participant enrollment by country

|  | **Participants receiving any LTP agent***  **(n = 35)** |
| --- | --- |
| **North America, n (%)**  United States | 19 (54.3) |
| **Europe, n (%)**  Austria  France  Germany  Italy  Romania  United Kingdom | 1 (2.9)  1 (2.9)  3 (8.6)  2 (5.7)  1 (2.9)  1 (2.9) |
| **Asia-Pacific, n (%)**  Japan | 7 (20.0) |

*LTP agents included berotralstat, lanadelumab, and C1INH replacement.
