# Supplementary material for: Sebetralstat for breakthrough attacks in patients with hereditary angioedema receiving long-term prophylaxis in KONFIDENT-S
Source: J Allergy Clin Immunol Glob. 2026 Jun 12;5(5):100750. doi: 10.1016/j.jacig.2026.100750 (PMC13355760; doi:10.1016/j.jacig.2026.100750)
Supplement: Supplementary Figure E1 [file mmc2.docx]

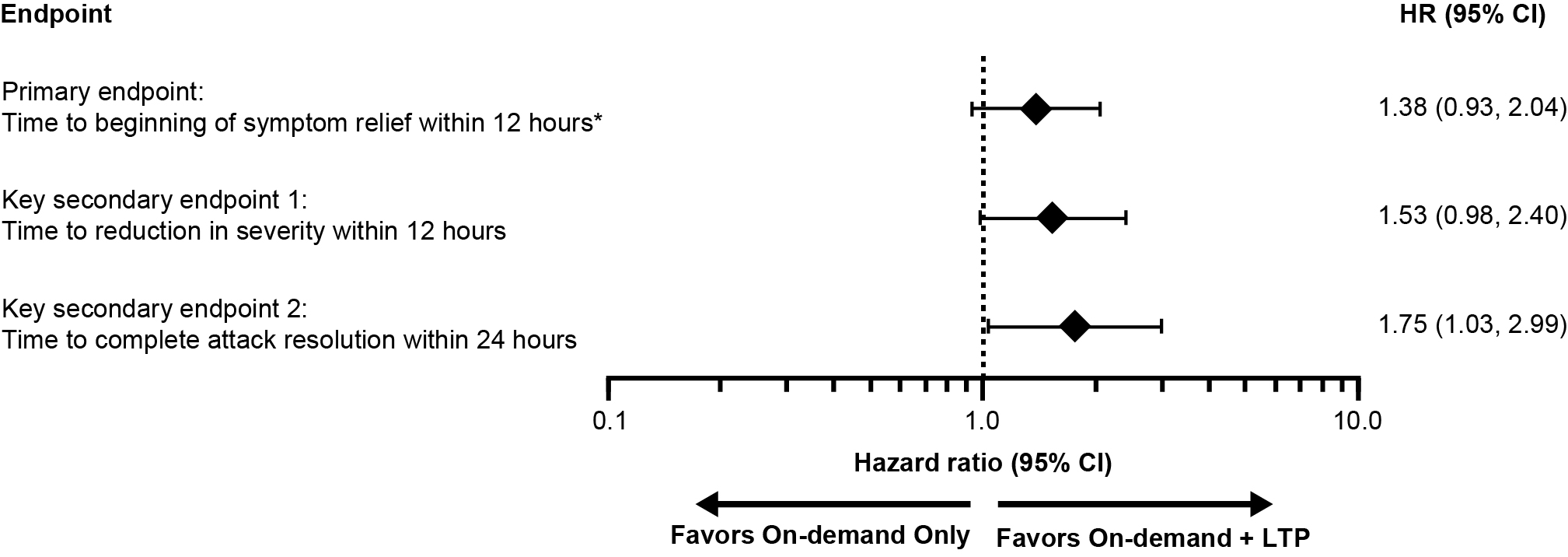


Figure E1. Hazard ratios (HRs) for primary and key secondary effectiveness endpoints comparing participants receiving LTP and participants receiving on-demand treatment only. *With missing data entries included. *CI,* confidence interval; *LTP,* long-term prophylaxis.
